# Effects of *Opuntia stricta* var. *dillenii* Extracts Obtained from Prickly Pear and an Industrial By-Product on Maturing Pre-Adipocytes

**DOI:** 10.3390/plants13212967

**Published:** 2024-10-24

**Authors:** Iván Gómez-López, Itziar Eseberri, Stéphanie Krisa, M. Pilar Cano, María P. Portillo

**Affiliations:** 1 Laboratory of Phytochemistry and Plant Food Functionality, Biotechnology and Food Microbiology Department, Institute of Food Science Research (CIAL) (CSIC-UAM), Nicolás Cabrera 9, 28049 Madrid, Spain; ivan.gomez@ehu.eus (I.G.-L.); mpilar.cano@csic.es (M.P.C.); 2 Nutrition and Obesity Group, Department of Nutrition and Food Science, Faculty of Pharmacy and Lucio Lascaray Research Center, University of the Basque Country (UPV/EHU), 01006 Vitoria-Gasteiz, Spain; itziar.eseberri@ehu.eus; 3 CIBERobn Physiopathology of Obesity and Nutrition, Institute of Health Carlos III (ISCIII), 01006 Vitoria-Gasteiz, Spain; 4 Bioaraba Health Research Institute, 01006 Vitoria-Gasteiz, Spain; 5 University of Bordeaux, INRAE, Bordeaux INP, Bordeaux Sciences Agro, UMR 1366, OENO, ISVV, F-33140 Villenave d’Ornon, France; stephanie.krisa@u-bordeaux.fr

**Keywords:** *Opuntia stricta* var. *dillenii*, betalains, phenolic compounds, 3T3-L1 pre-adipocytes, adipogenesis, triacylglycerol, adiponectin

## Abstract

*Opuntia stricta* var. *dillenii*, a member of the Cactaceae family, produces a fruit known as prickly pear. This fruit is rich in bioactive compounds, including betalains and phenolic compounds, which play an important role in health promotion due to their antioxidant and anti-inflammatory properties. This study aims to investigate the impact of prickly pear extracts obtained from the whole fruit, peel, pulp, and an industrial by-product (bagasse) on the differentiation of 3T3-L1 pre-adipocytes. During the differentiation process, 3T3-L1 pre-adipocytes were treated with prickly pear extracts at concentrations ranging from 10 to 100 μg/mL from day 0 to day 8 post-induction. Moreover, the potential mechanisms justifying the observed effects were assessed by RT-PCR. All extracts led to an increase in both triacylglycerol accumulation and cell number. In conclusion, the analysed extracts demonstrated adipogenic effects in 3T3-L1 maturing pre-adipocytes by increasing the expression of the *c/ebp-β*, *srebf-1*, and *c/ebp-α* genes. Additionally, a potential anti-inflammatory effect was observed through the upregulation of adiponectin.

## 1. Introduction

According to the European Health Survey, in Spain, 16.5% of adult men, 15.5% of adult women, and around 10% of children were obese in 2020 [1]. The prevalence of obesity is increasing, and it is projected that by 2035, it will affect 35% of all women and 39% of all men in Europe [2]. Obesity is one of the leading causes of premature mortality and disability worldwide. It is characterised by abnormal or excessive fat accumulation in adipose tissue, leading to heightened stress and dysfunction within this tissue. Furthermore, this increased fat accumulation contributes to the development of obesity-related co-morbidities, including cardiovascular diseases, type 2 diabetes, musculoskeletal disorders and certain cancers [3].

The expansion of adipose tissue (commonly known as body fat) involves various complex processes influenced by factors such as diet, physical activity, genetics, hormones and metabolic regulations. When caloric intake surpasses energy expenditure, energy is stored as fat in the adipocyte cells. Two distinct processes may contribute to the expansion of adipose tissue: the increase in the number of adipocytes within the tissue (hyperplasia), arising from the differentiation of cell precursors to generate new adipocytes—a phenomenon known as adipogenesis—and the enlargement of mature adipocytes (hypertrophy). The proliferation of hypertrophic adipose tissue has been linked to metabolic dysfunction, characterised by a pro-inflammatory profile and insulin resistance [4].

Due to its high prevalence, the prevention and treatment of obesity is a top priority for health systems. Bioactive compounds, which are naturally occurring substances in foods, have been the focus of research for their potential role in preventing or managing obesity. These compounds can exert various physiological effects on the body, including anti-inflammatory, antioxidant, and metabolism-regulating properties [5,6].

**Figure 1:**
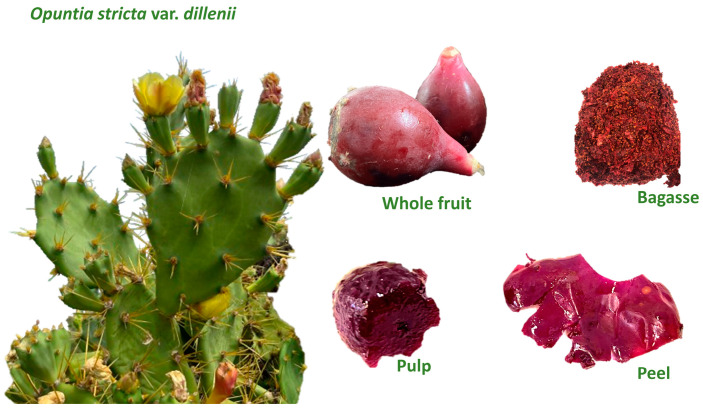
*Opuntia stricta* var. *dillenii* cactus and prickly pear tissues (whole fruit, peel and pulp) and by-product (bagasse) from the Canary Islands, Spain.

In recent years, prickly pear (*Opuntia*) has garnered considerable attention due to its promising medicinal and nutritional properties, which have the potential to offer positive benefits for diabetes, obesity and inflammatory diseases [7,8]. This fruit belongs to the most abundant family among the *Cactaceae*, encompassing approximately 300 different varieties. It is rich in betalains, which are nitrogen-based water-soluble pigments synthesised as secondary metabolites and accumulated in vacuoles in the plant cell cytoplasm, imparting vibrant colours such as purple (from betacyanin compounds) and yellow (from betaxanthin compounds) to the plant. In the Canary Islands (Spain), several interesting varieties of *Opuntia*, such as *Opuntia stricta* var*. dillenii*, grow wild without any special requirements. Its consumption is local, and only a limited number of scientific works focusing on this variety have been published to date. This fruit has a distinctive dark purple colour (Figure 1), which is attributed to its richness in betacyanins, primarily betanin, neobetanin and phylocactin [9].

Betalains have attracted increased attention due to their potential health benefits, which include their anti-inflammatory, antibacterial, antioxidant and lipid-lowering effects [10,11]. Previous studies from our group have also revealed that the fruits of *Opuntia stricta* var*. dillenii* are rich in some phenolic compounds, such as piscidic acid and flavonoids, particularly isorhamnetin glucosides [9]. These phenolic compounds from *Opuntia* have been reported to exhibit antioxidant and anti-inflammatory activities [12].

In this context, our hypothesis is that extracts of *Opuntia stricta* var. *dillenii*’s fruit, due to their bioactive compounds, are able to regulate the adipogenic process and reduce lipid accumulation in maturing pre-adipocytes. Thus, this current study aims to investigate the impact of *Opuntia stricta* var. *dillenii* extracts obtained from the whole fruit, the peel and the pulp, as well as from an industrial by-product (bagasse), all rich in betalains and phenolic compounds, on 3T3-L1 maturing pre-adipocytes. Our objective is to identify the most sustainable starting material with proven biological activities. Moreover, the potential mechanisms involved in the observed effects are also analysed.

## 2. Results

### 2.1. Effects on Cell Viability and Triacylglycerol Content

**Figure 2:**
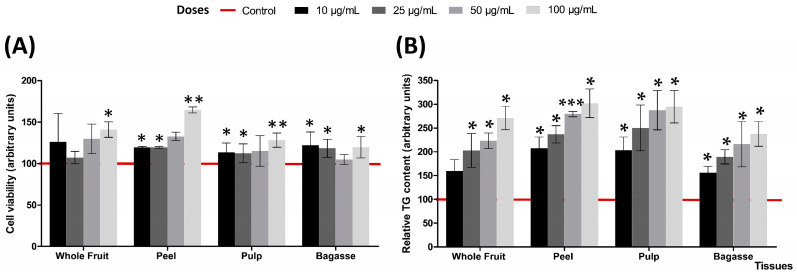
Effects of *Opuntia stricta* var. *dillenii* tissues (whole fruit, peel, pulp and bagasse) extracts at 10, 25, 50 and 100 µg/mL on (**A**) cell viability (%) and (**B**) triacylglycerol content (%) of 3T3-L1 maturing pre-adipocytes treated from day 0 to day 8. Values are means ± SEM. Comparison between each tissue extract dose and the control was analysed by Student’s *t*-test. The asterisks represent differences versus the controls (* *p* < 0.05; ** *p* < 0.01; *** *p* < 0.001).

No cytotoxic effects were observed in the cells after incubation with the *Opuntia stricta* var*. dillenii* extracts at doses of 10, 25, 50, or 100 µg/mL from day 0 to day 8 (the adipogenic period). Conversely, it is noteworthy that all the *Opuntia stricta* var*. dillenii* extracts at the highest dose (100 µg/mL) significantly enhanced cell viability compared to the control cells (Figure 2A). Moreover, doses of 10 µg/mL and 25 µg/mL of the peel, pulp and bagasse also exhibited a significant increase in cell proliferation. Additionally, peel doses of 50 µg/mL showed a tendency towards increased values.

Triacylglycerol accumulation throughout the adipogenic process showed a significant boost with all the extracts at the four doses utilised compared to the non-treated cells (controls). Notably, the only exception was the whole fruit extract at a dose of 10 µg/mL, which exhibited a tendency (*p <* 0.1) towards higher values (+56%) (Figure 2B and Table 1). It is noteworthy that the quantity of accumulated triacylglycerols in cells exhibited an escalating trend corresponding to the dose of the extracts employed (Figure 2B).

### 2.2. Effect on Adipogenic Expression

To understand the mechanisms underlying the observed effects, we analysed *adiponectin* and the expression of the genes involved in the adipogenic process (*c/ebp-α*, *c/ebp*-*β*, *srebf-1* and *ppar-γ*), some of which are considered markers of adipocyte maturation (*acc*, *atgl* and *hsl*). This analysis was performed on cells treated with the highest dose (100 µg/mL) of each extract (whole fruit, peel, pulp and bagasse).

**Figure 3:**
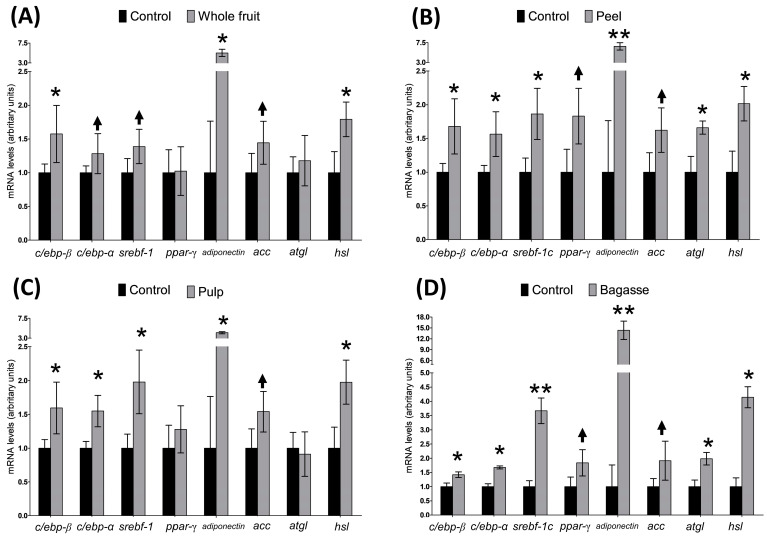
Effects of *Opuntia stricta* var. *dillenii* extracts at a dose of 100 µg/mL from (**A**) whole fruit, (**B**) peel, (**C**) pulp (**D**) and bagasse on gene expression of *c/ebp-β*, *c/ebp-α*, *srebf-1c*, *ppar-γ*, *adiponectin*, *atgl*, *acc* and *hsl* in 3T3-L1 maturing pre-adipocytes treated from day 0 to day 8. Values are means ± SEM. Comparisons between each tissue extract dose and the control were analysed by Student’s *t*-test. The asterisks represent differences versus the controls (* *p* < 0.05; ** *p* < 0.01). Arrows mean a tendency (*p* < 0.10).

The expression of the *c/ebp-β* gene exhibited a significant increase following exposure to the four extracts (Figure 3). Furthermore, extracts from the peel, pulp and bagasse significantly elevated the gene expression of *srebf-1* and *c/ebp-α* in maturing pre-adipocytes. In contrast, cells treated with the whole fruit extract showed only a tendency towards higher values. For *ppar-*γ, a trend towards enhanced values (*p* < 0.1) was evident in the pre-adipocytes treated with extracts from peel or bagasse (Figure 3).

In terms of the expression of the genes considered markers of adipocyte maturation, *hsl* showed a significant increase across all treatments, while *atgl* expression was notably enhanced only in the cells treated with the extracts from peel and bagasse (Figure 3). Additionally, the expression of *acc* displayed a noticeable trend in all treated cells. Interestingly, the *adiponectin* levels surged following incubation with each extract.

## 3. Discussion

As explained in the Introduction section, *Opuntia stricta* var*. dillenii*, which grows wild in the Canary Islands, has been scarcely studied to date. In fact, no data are available in the literature concerning its effects on adipocytes. Consequently, the data submitted in the present study represent a significant contribution to the existing knowledge.

All the extracts at the four tested doses resulted in a significant increase in lipid accumulation of maturing pre-adipocytes. Since there is a lack of available data in the literature regarding the effects of *Opuntia stricta* var*. dillenii* fruit extracts on adipocytes, we are unable to directly compare our results with other studies. Therefore, we will compare our findings with those obtained from other *Opuntia* species and different parts of the plant. In a study utilising cladode powder from two *Opuntia* species (*Streptacantha* and *ficus-indica*), the authors observed that a 10-day treatment of 3T3-F442A maturing pre-adipocytes with the extracts resulted in reduced lipid accumulation in the cells [13]. On the other hand, in a previous study conducted by our group, we investigated the effects of six extracts derived from the peel and the pulp of the fruit from three varieties of *Opuntia ficus-indica* L. Mill. (Pelota, Colorada and Sanguinos) on 3T3-L1 maturing and mature adipocytes [11]. In that research, we noted enhanced triacylglycerol accumulation in the pre-adipocytes treated with extracts from Pelota pulp, Sanguinos peel and Colorada pulp. In contrast, extracts from Sanguinos pulp and Colorada peel at the lowest doses reduced lipid accumulation in cells. It is important to consider that different *Opuntia* species, varieties and tissues were used in the three experiments, potentially accounting for the observed variations.

Relative to the aforementioned, Gómez-Maqueo et al. [14] reported different antioxidant activities for the extracts of *Opuntia stricta* var*. dillenii* and *Opuntia ficus-indica* L. Mill., which were attributed to their different bioactive compound profiles. For instance, *Opuntia stricta* var*. dillenii* contains higher amounts of betalains and phenolic compounds such as Neobetanin, Phyllocactin, … [9]. Moreover, El-Hawary and colleagues recently summarised the different bioactive compounds present in the cladode, fruit peel and fruit pulp of *Opuntia ficus-indica*, observing highly significant differences among them [15]. Thus, the different compound profiles of the extracts could explain, at least in part, the varying potential effects of *Opuntia* on lipid accumulation in maturing pre-adipocytes. *Opuntia stricta* var*. dillenii*, prickly pear fruit peels contain more total phenolic compounds (phenolic acids and flavonoids) compared to the fruit pulp, with piscid acid and isorhamnetin glucoxyl-rhamnosyl-pentoside (IG2) being the most abundant, with 2.33 ± 0.33 mg/g dry weight and 0.52 ± 0.02 mg/g dry weight, respectively (Tables S1 and S2). The pulp is richer in all the identified betalains, with 12.78 ± 0.48 mg of the sum/g dry weight (Tables S1 and S2).

To confirm the non-toxicity of the tested extracts, we conducted a cell viability assay using DNA staining to quantify the cell numbers. Interestingly, the cell count increased in the treated cells compared to the controls. Specifically, at the highest dose (100 µg/mL), all the extracts demonstrated the ability to enhance cell numbers (Figure 2A). This is not the first instance where an increase in cell viability has been observed following treatment of pre-adipocytes with plant or fruit extracts. In the aforementioned study by Eseberri et al. [11], cell viability increased after treatment with certain doses of peel and pulp extracts from the three varieties of *Opuntia ficus-indica*. Similarly, in a study conducted with two *Opuntia* species (*Streptacantha* and *ficus-indica*) using cladode powders, the authors found that a 10-day treatment with the extracts boosted the cell numbers in maturing 3T3-F442A adipocytes [13]. Treatments with other extracts containing bioactive compounds have also demonstrated an increase in cell proliferation; Kim et al. [16] reported an enhanced 3T3-L1 pre-adipocyte proliferation after treatment with low concentrations of curcumin. Additionally, a significant boost of live cells was observed after a 9-day treatment of 3T3-L1 pre-adipocytes with four doses of a citrus peel extract [17]. Based on these findings, a proliferative effect of the *Opuntia* extracts can be suggested.

**Figure 4:**
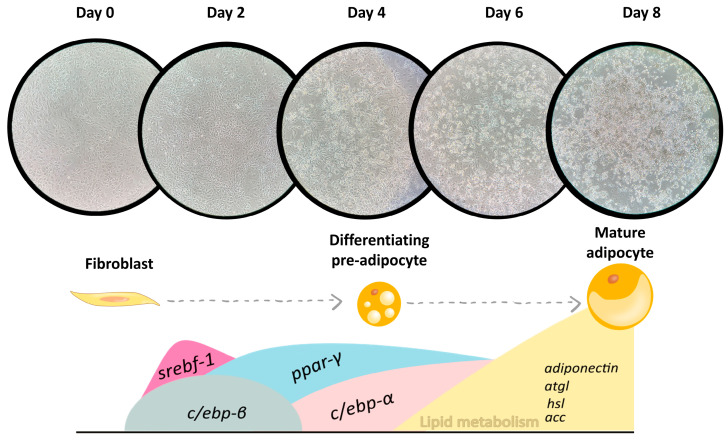
Cell differentiation and lipid accumulation in 3T3-L1 pre-adipocytes and gene expression profile adipogenesis. *c/ebp-β:* CCAAT/enhancer-binding protein beta; *c/ebp-α:* CCAAT/enhancer-binding protein alpha; *srebf-1c:* SREBP cleavage-activating protein 1, c isoform; *ppar-γ:* Peroxisome Proliferator-Activated Receptor gamma*; atgl:* Adipose triglyceride lipase; *acc:* Acetyl coenzyme A carboxylase; and *hsl:* Hormone-sensitive lipase.

Adipogenesis is the biological process that allows precursor cells to differentiate into adipocytes, which involves several stages and molecular events that culminate in the formation of mature adipocytes. This process can be divided into two main steps: during the first one, fibroblast differentiation into the adipocyte lineage takes place, leading to the formation of pre-adipocytes; during the second step, pre-adipocytes accumulate triacylglycerols in the cytosol, resulting in mature adipocytes (Figure 4). Substances with the potential to decrease adipogenesis are frequently regarded as promising candidates for anti-obesity interventions [18,19]. However, adipogenesis is now emerging as a potential therapeutic strategy to enhance adipose tissue health and counteract the negative metabolic consequences associated with adipocyte hypertrophy [20,21]. Hyperplasia-induced adipose tissue growth is considered protective against the health risks associated with obesity [22]. It facilitates proper vascularisation of the tissue, and, in conjunction with a reduced number of hypertrophic adipocytes, it leads to higher insulin sensitivity and lower levels of pro-inflammatory cytokines [4,21]. Interestingly, adipocytes efficiently accumulate lipids, thereby preventing toxic ectopic lipid accumulation (lipotoxicity) in other organs and tissues, such as skeletal muscle as well as the liver and heart [4]. This capacity strongly correlates with preserved metabolic functions across associated pathologies. On the contrary, failure in adipocyte differentiation has been proposed as one of the contributing factors to type 2 diabetes [4]. Consequently, it has been suggested that the ability to recruit new adipocytes through adipogenesis is critical for both healthy adipose tissue expansion and systemic metabolic health in the setting of caloric excess [4,23]. In this context, identifying novel adipogenesis regulators that promote hyperplasia may lead to effective therapies for obesity-induced metabolic disorders. Therefore, the effects induced by the fruit extracts from *Opuntia stricta* var*. dillenii* on cell viability and triacylglycerol content can be considered beneficial for obesity management. In fact, in a further in vivo study carried out in rats on an obesogenic diet, we tested the effects of an *Opuntia stricta* var. *dillenii* peel extract. Our findings revealed that rats supplemented with this extract exhibited reduced visceral adipose tissue sizes in comparison with the control group (rats fed the same obesogenic diet without *Opuntia* supplementation).

The adipogenic process is initiated by several proteins; among them, C/EBP-β and C/EBP-δ, two transcription factors belonging to the co-activator CCAAT/enhancer-binding family, appear to be particularly important in this first phase [24]. Another transcription factor involved in the early differentiation state is SREBP cleavage-activating protein 1, c isoform (SREBP-1c), which plays a significant role in the induction of the late adipogenic phase (Figure 4). The results obtained in this present study show that the incubation of pre-adipocytes with the whole fruit extract led to an upregulation in the expression of *c/ebp-β* and a tendency towards an elevated value of *srebf-1* (Figure 3). The peel, pulp and bagasse extracts induced an increase in gene expression of both *c/ebp-β* and *srebf-1* (Figure 3). With these data, we can conclude that all the tested extracts are able to stimulate the first phase of adipogenesis.

The over-expression of C/EBP-*β* and SREBP-1c induces and accelerates adipogenesis. by promoting the expression of transcription factors in the late adipogenic step, such as PPAR-γ and C/EBP-α [4,25,26,27]. In this present study, treatment with all the extracts significantly increased or tended to increase the expression of *c/ebp-β*, *srebf-1* and *c/ebp-α*, but not that of *ppar-γ*. Although no data are available to directly compare these results with other studies, and since the bioactivity of the tested extracts can be confidently attributed to their bioactive compound profile (mainly based on betalains, phenolic acids and flavonoids), we conducted a search in the scientific literature to determine whether similar results have been reported. Some studies have shown that flavonoids exhibit a pro-adipogenic effect by increasing the gene expression of PPAR-γ, C/EBP-α, and adiponectin in 3T3-L1 adipocytes [28,29]. As mentioned earlier in this study, the observed increase in *c/ebp-*α mRNA levels was not fully accompanied by a significant stimulation of *ppar-γ*, although a tendency was observed after treatment with the peel and bagasse extracts (Figure 3). One of the most significant downstream effects of PPAR-γ is the activation of C/EBP-α [30]. While it is assumed that C/EBP-α requires PPAR-γ for its full activation, it has been demonstrated that C/EBP-α alone is sufficient to promote adipocyte differentiation, as Freytag and co-workers showed after retroviral infection and DNA transfection of cultured fibroblasts [31]. This apparent discrepancy in the expression pattern of both genes in this study has also been reported by other authors. In this context, Chyau et al. [32] analysed the effect of 100 µM of rutin, kaempferol and betanin in lipid accumulation and the gene expression of PPAR-γ, C/EBP-α and SREBP-1c in maturing 3T3-L1 pre-adipocytes. They observed that all three molecules reduced lipid accumulation in cells. Specifically, kaempferol lowered the expression levels of PPAR-γ, C/EBP-α and SREBP-1c, whereas rutin reduced that of C/EBP-α and SREBP-1c without affecting PPAR-γ. Betanin only decreased the expression of SREBP-1c. In another study, Sung et al. [33] investigated the antiadipogenic expression of benzo[b]furan derivatives, and they found that treatment with 5 µM of 2-(3′-methoxy-4′-hydroxy-phenyl)-6-(3-hydroxypropyl)-5-methoxy-benzo[b]furan lowered the lipid content in 3T3-L1 pre-adipocytes. This reduction was associated with decreased levels of C/EBP-α, while PPAR-γ remained unaffected. In a follow-up study, the same group explored the role of Cyclin C on adipogenesis regulation. They observed that Cyclin C was essential for the activation of C/EBP-α but not for that of PPAR-γ, which suggests a very complex interaction among proteins that drive the adipogenic process [34]. In other words, while it has conventionally been assumed that simultaneous activation of both C/EBP-α and PPAR-γ is necessary for pre-adipocyte differentiation, data in the literature indicate that this does not always occur. Thus, our findings demonstrate that the bioactive compounds present in the *Opuntia stricta* var*. dillenii* extracts modulate *c/ebp-*α expression without clear activation of *ppar-γ*, promoting effective adipocyte differentiation.

At the end of the differentiation process, mature adipocytes express characteristic markers associated with maturity, including the expression of *adiponectin*, *atgl*, *acc* and *hsl.* The expression of these genes was not uniformly increased by the *Opuntia stricta* var*. dillenii* extracts (Figure 3). Specifically, all extracts significantly increased the *adiponectin* and *hsl* mRNA levels and tended to enhance those of *acc*. However, only the peel and bagasse extracts increased the *atgl* gene expression. Considering the anti-inflammatory action of *adiponectin* and its positive effects on insulin sensitivity [35], the significant increase in its expression induced by the *Opuntia stricta* var*. dillenii* extracts is remarkable because it indicates the promotion of adipocytes with a healthier metabolic profile.

## 4. Materials and Methods

### 4.1. Plant Material and Preparation of Extracts

The *Opuntia stricta* var*. dillenii* prickly pears used in this study were harvested in September 2020 in Tenerife (The Canary Islands, Spain) at an elevation of 209 m above sea level. After washing, the fruits were chosen based on their size, colour and ripeness; damaged products were discarded. The fruits were manually processed into three main components: whole fruit tissue, pulps (mesocarp) and peels (endocarp and exocarp), as illustrated in Figure 1. Additionally, bagasse, a by-product of prickly pears obtained from the jam industry, was sourced from Bernardo’s company in Lanzarote, Spain. All samples were cut into small cubes (1 × 1 cm), immediately frozen with liquid nitrogen and freeze-dried for five days at—45 °C and 1.3 × 10^–3^ MPa (LyoBeta 15, Azbil Telstar, S.L., Terrasa, Spain). The freeze-dried tissues were pulverised (Grindomix GM200, Haan, Germany) to a fine particle size (<2 mm) following seed removal.

From the pulverised freeze-dried *Opuntia stricta* var*. dillenii* tissues (whole fruit, pulp, peel) and by-product (bagasse), extracts rich in betalains and phenolic compounds were obtained as previously documented [9]. In summary, 1 g of each freeze-dried tissue was subjected to extraction using a 5 mL mixture of ethanol and water (1:1, *v*/*v*). This extraction procedure was repeated twice using 3 mL of ethanol and water (1:1, *v*/*v*) in each iteration. A final extraction was carried out using 3 mL of pure ethanol. The solvents in the supernatants were then reduced to a minimal volume using a rotary evaporator (Buchi, Flawil, Switzerland) set at 25 °C. Aliquots of each tissue extract were freeze-dried and stored at −20 °C until further use.

### 4.2. HPLC-DAD Characterisation: Betalains and Phenolic Compounds

Following the methodology previously reported by our research group [9], the identification and quantification of betalains and phenolic compounds in the *Opuntia stricta* var*. dillenii* extracts were performed simultaneously using high-performance liquid chromatography (HPLC-DAD).

An Agilent 1200 Series HPLC system (Agilent Technologies, Santa Clara, CA, USA) equipped with a Zorbax SB-C18 reverse-phase column (250 × 4.6 mm i.d., 5 μm particle size; Agilent) maintained at 25 °C was used. Ultrapure water with 1% formic acid (*v*/*v*) and methanol (99.8% LC-MS) with 1% formic acid (*v*/*v*) were used as phases A and B in a gradient elution over 70 min. The injection volume selected for the analysis was 20 μL, and the flow rate was determined at 0.8 mL/min. The UV–visible photodiode array detector was set at four wavelengths: phenolic acids were detected at 280 nm, flavonoids at 370 nm, betaxanthins at 480 nm and betacyanins at 535 nm.

To validate the chemical composition of each bioactive compound, further analyses were conducted using the HPLC-DAD-MS/QTOF and HPLC-DAD-ESI/MS techniques. The compounds were identified by comparing their UV/vis maximum absorption (λmax), mass spectral data (*m*/*z*) and retention times (rt) with those of commercial, semi-synthesised or purified standards.

Quantitation of the most abundant betalains, piscidic acid and isorhamnetin glycosides was determined using the calibration curves of the corresponding isolated standards. The complete description of the UV–vis and mass spectroscopy characteristics of all individual betalains and phenolic compounds found in the *Opuntia stricta* var*. dillenii* extracts was documented in a previous work [9].

The characterisation of the used extracts has recently been described in work aimed to analyse the anti-obesity effect of the extracts in 3T3-L1 mature adipocytes [36]. In summary, the extracts rich in betalains and phenolic compounds result in the fruit exhibiting a vibrant purple colour, as depicted in Figure 1, attributable to the presence of compounds known as betacyanins. The main compounds within betacyanins in *Opuntia stricta* var*. dillenii* tissues are betanin, isobetanin, 5″-O-E-sinapoyl-2′-apyosil-phyllocactin and neobetanin. As a general observation, the betalain pattern was consistently evident across the *Opuntia stricta* var*. dillenii* prickly pear tissues and by-products, with higher pigment levels observed in the fruit tissues compared to bagasse. In terms of the phenolic compounds, *Opuntia* prickly pear can be classified into two predominant families: phenolic acids and flavonoids. Piscidic acid stands out as the primary component of phenolic acids, a compound seldom found in nature and restricted to certain plants [7]. Regarding flavonoids, the most prevalent was isorhamnetin-glucosyl-rhamnosyl-pentoside (IG2). This fruit is also a source of quercetin glycosides, such as Quercetin-3-*O*-rhamnosyl-rutinoside (QG3), Quercetin glycoside (QG1) Quercetin hexosyl pentosyl rhamnoside and Quercetin glycoside (QG2) Quercetin hexose pentoside.

The descriptive data, chromatograms obtained from HPLC-DAD, and the most abundantly identified and quantified compounds in the extracts from the whole fruit, peel, pulp, and bagasse of *Opuntia stricta* var*. dillenii* are presented in Tables S1 and S2 according to Figure S1 (Supplementary Materials), as detailed in the publication [36].

### 4.3. Pre-Adipocyte Cell Experimental Design

The 3T3-L1 pre-adipocytes were provided by the American Type Culture Collection (Manassas, VA, USA) and cultured in Dulbecco’s Modified Eagle Medium (DMEM) supplemented with 10% foetal bovine serum (FBS) and 1% penicillin/streptomycin (PS; 10,000 U/mL). Two days following confluence, the pre-adipocytes were cultured in a differentiation induction medium composed of DMEM with 10% FBS and 1% PS, 10 g/mL of insulin, 0.5 mM isobutylmethylxanthine (IBMX), and 1 mM dexamethasone for 48 h (day 0). Subsequently, the differentiation medium was replaced with FBS/DMEM/PS supplemented with 10 µg/mL of insulin for two days. From day 4 to day 8, the differentiation medium consisted of FBS/DMEM/PS enriched with 0.2 g/mL of insulin. The cells were maintained at 37 °C in a humidified 5% CO_2_ atmosphere.

Maturing pre-adipocytes, seeded in 6-well plates, were incubated with extracts from *Opuntia stricta* var*. dillenii*, specifically the whole fruit (WF), peel (PE), pulp (PU) or bagasse (BA), at concentrations of 10, 25, 50 or 100 µg/mL (diluted in milli-Q water) throughout the adipogenic phase from day 0 to day 8 of differentiation. The medium was refreshed every 48 h (two days). On day 8, the cells were harvested for triacylglycerol measurement and gene expression determination. Each experiment was conducted in triplicate.

### 4.4. Cell Viability Assay

Cells were seeded onto 96-well plates and maintained under the same conditions as described in Section 4.3, including treatment. Following the protocol described by Gilles et al. [37], cell viability was assessed through crystal violet DNA staining for the viable cells. To summarise, the cells were washed with sterile phosphate-buffered saline (PBS) and fixed with formaldehyde (3.7%). Crystal violet (0.25%) was applied for DNA staining, with the cells being incubated in the absence of light for 30 min. The resulting crystals were dissolved in acetic acid (33%), and the absorbance was measured at 590 nm using an iMark microplate reader (Bio-Rad, Hercules, CA, USA). The absorbance recorded for each well was directly proportional to the cell density, and the results are presented in arbitrary units.

### 4.5. Measurement of Triacylglycerol Content

Following the cell treatment, the medium was removed, and the adipocytes were thoroughly washed with PBS. The cells were then harvested using 300 µL of buffer comprising Tris-HCl at pH 7.4, 150 mM NaCl, and 1 mM EDTA with protease inhibitors (100 mM phenylmethylsulfonyl fluoride and 100 mM iodoacetamide). Subsequently, using the Branson Digital Sonifier SFX 550 (Emerson Electric Co., St. Louis, MO, USA) with a 2 mm diameter ultrasound microprobe (Biogen Scientific S.L., Madrid, Spain), the cell samples were disrupted. The triacylglycerol content (mg/mL) was measured using the Infinity triacylglycerol reagent (Thermo Scientific, Rockford, IL, USA). The protein content of each well was used to standardise the lipid content of the cells and determined using the method described by Bradford et al. [38]. The results are expressed as mg of triacylglycerols per mg of protein and presented in arbitrary units.

### 4.6. RNA Preparation and Quantitative Real-Time PCR

RNA was extracted from cells using 700 µL of Trizol (Invitrogen, Carlsbad, CA, USA) per well. The RNA 6000 Nano Assay (Thermo Scientific, Wilmington, DE, USA) was used to verify and quantify the integrity of the RNA extracted from all samples. Subsequently, the RNA samples underwent treatment with the DNase I kit (Applied Biosystems, Foster City, CA, USA) to eliminate potential contamination with genomic DNA. For the reverse transcription of 1.5 µg of total RNA from each sample into first-strand complementary DNA (cDNA), the iScript cDNA Synthesis Kit (Bio-Rad, Hercules, CA, USA) was utilised. For the reaction, samples were incubated at 25 °C for the initial 10 min, followed by incubation at 37 °C for 120 min, concluding at 85 °C for 5 min.

The mRNA levels of the following CCAAT/enhancer-binding proteins are as follows: *α* and *β* (*c/ebp*); Sterol Regulatory Element-Binding Transcription Factor 1 (*srebf-1)*; Peroxisome Proliferator-Activated Receptor gamma (*ppar-γ*); Adipose triacylglycerol lipase (*atgl*); Acetyl coenzyme A carboxylase (*acc*); Hormone-sensitive lipase (*hsl*); Cyclin-Dependent Kinase Inhibitor 1A (*cdkn1a*); Cyclin-Dependent Kinase Inhibitor 1B (*cdkn1b*); there were quantified using an iCycler-MyiQ Real-Time PCR Detection System (BioRad, Hercules, CA, USA). *β*-actin served as the reference gene (housekeeping). The PCR reagent mixture included a 4.75 μL aliquot of each diluted cDNA, SYBR Green Master Mix (Applied Biosystems, Foster City, CA, USA), along with upstream and downstream primers at a concentration of 5 nM. The primer sequences and PCR conditions for the specific commercially synthesised primers are presented in Table 2. All mRNA levels were normalised to the values of *β*-actin, and the results were expressed as fold changes in the threshold cycle (Ct) value relative to the controls using the 2^−ΔΔCt^ method [39].

### 4.7. Statistical Analysis

Data were analysed using SPSS Statistics software version 26.0 (IBM Corp., Armonk, NY, USA) and expressed as the mean ± standard error of the mean (SEM). The impact of each extract on the triacylglycerol levels, gene expressions and cell survival was assessed by comparing each treated cell group with the control cells using the Student’s *t-*test. The threshold for statistical significance was set at *p <* 0.05.

## 5. Conclusions

In conclusion, the present results demonstrate that the extracts enhance adipogenesis in 3T3-L1 cells, as evidenced by a higher triacylglycerol accumulation and upregulation of key adipogenic genes. Given the potential of pro-adipogenic compounds in addressing obesity-associated co-morbidities like type 2 diabetes, these extracts may be valuable for future therapeutic development. Furthermore, the upregulation of adiponectin gene expression suggests a potential anti-inflammatory effect of the extracts.

Our findings indicate that this fruit can serve as a promising starting material for producing extracts rich in betalains and phenolic compounds, with potential applications in the management of obesity and its associated co-morbidities. To select the most suitable extracts, it is worth considering that, in alignment with the principles of the 2030 Agenda for Sustainable Development, the use of non-edible fractions such as peel and bagasse has emerged as a compelling option. Incorporating these non-edible parts of the fruit into valuable extracts not only maximises resource utilisation but also minimises waste, contributing to broader global efforts to achieve sustainable development goals. This approach benefits the environment by reducing agricultural waste and holds the potential to create economic value from parts of the fruit that would otherwise be discarded. This dual benefit aligns with the growing emphasis on integrating health solutions with sustainable practices, providing a comprehensive view of how these extracts could be used in the future. In light of our findings and considering that both the peel and the bagasse extracts exhibit interesting effects on adipogenesis, but only the peel extract effectively prevents triacylglycerol accumulation in mature adipocytes, we propose the *Opuntia stricta* var*. dillenii* peel as the optimal starting material for future in vivo studies.

## Figures and Tables

**Table 1:** Effects of *Opuntia stricta* var. *dillenii* tissue (whole fruit, peel, pulp and bagasse) extracts at 10, 25, 50 and 100 µg/mL on triacylglycerol content (%) of 3T3-L1 maturing pre-adipocytes treated from day 0 to day 8. Values are means ± SEM.

Triacylglycerol Content (%)				
Doses	Tissue			
	Whole Fruit	Peel	Pulp	Bagasse
10 µg/mL	155.71 ± 23.69	207.36 ± 23.80 *	203.47 ± 27.85 *	157.00 ± 13.84 *
25 µg/mL	202.81 ± 35.78 *	210.90 ± 18.19 *	250.43 ± 48.16 *	189.62 ± 14.96 *
50 µg/mL	223.19 ± 16.35 *	279.35 ± 5.63 ***	287.43 ± 41.29 *	216.57 ± 47.59 *
100 µg/mL	271.10 ± 24.68 *	302.06 ± 4.80 *	294.78 ± 34.02 *	237.69 ± 26.02 *

Control represents 100% of accumulation. The results were expressed as mean ± standard deviation (*n* = 3). Asterisks indicate significant differences between different *Opuntia stricta* var. *dillenii* tissues and control (* *p* < 0.05; *** *p* < 0.001).

**Table 2:** Primer sequences for real-time quantification PCR amplification of each studied gene by SYBR^®^ Green.

Gene	Sense Primer	Antisense Primer	Annealing (°C)
*c/ebp-β*	5′-CAA GCT GAG CGA CGA GTC CA-3′	5′-CAG CTG CTC CAC CTT CTT CT-3′	56.5
*c/ebp-α*	5′-TGG ACA AGA ACA GCA ACG AG-3′	5′-TCA CTG GTC AAC TCC AGC AC-3′	56.5
*srebf-1*	5′-GCT GTT GGC ATC CTG CTA TC-3′	5′-TAG CTG GAA GTG ACG GTG GT-3′	57.6
*ppar-γ*	5′-TCG CTG ATG CAC TGC CTA TG-3′	5′-GAG AGG TCC ACA GAG CTG-3′	60.0
*adiponectin*	5′-GAC GAC ACC AAA AGG GCT CA-3′	5′-GAG TGC CAT CTC TGC CAC CA-3′	60.0
*atgl*	5′-GAG CTT CGC GTC ACC ACC-3′	5′-CAC ATC TCT CGG AGG ACC A-3′	58.5
*acc*	5′-GGA CCA CTG CAT GGA ATG TTA-3′	5′-TGA GTG ACT GCC GAA ACA TCT-3′	57.8
*hsl*	5′-GGT GAC ACT CGC AGA AGA CAA TA-3′	5′-GCC GCC GTG CTG TCT CT-3′	60.0

*c/ebp-β*: CCAAT/enhancer-binding protein beta; *c/ebp-α*: CCAAT/enhancer-binding protein alpha; *srebf-1*: SREBP cleavage-activating protein 1, c isoform*; ppar-γ*: Peroxisome Proliferator-Activated Receptor gamma; *atgl*: Adipose triglyceride lipase; *acc*: Acetyl coenzyme A carboxylase; *hsl*: Hormone-sensitive lipase.

## Data Availability

Data are contained within the article and Supplementary Materials.

## Supplementary Materials

The following supporting information can be downloaded at: https://www.mdpi.com/article/10.3390/plants13212967/s1, Figure S1: HPLC-DAD chromatogram of betalains (at 480 and 535 nm) and phenolic compounds (at 280 and 370 nm) in *Opuntia stricta* var. *dillenii* from (a) whole fruit, (b) peel, (c) pulp and (d) bagasse. Numbers correspond to the identified compounds indicated in Table S1 according to Gómez-Lopez et al. [9], Table S1: HPLC retention time (Rt), maximum absorption (λmax) and *m*/*z* of the identified major bioactive compounds from *Opuntia stricta* var. *dillenii* based on Gómez-Lopez et al. [9], Table S2: Bioactive compounds content (mg/g dry weight) of the major betalains and polyphenols in peel, pulp and whole fruit and by-product (bagasse) from *Opuntia stricta* var. *dillenii* prickly pear based on Gómez-Lopez et al. [9].
