# Supplementary material for: Effects of Opuntia stricta var. dillenii Extracts Obtained from Prickly Pear and an Industrial By-Product on Maturing Pre-Adipocytes
Source: Plants (Basel). 2024 Oct 24;13(21):2967. doi: 10.3390/plants13212967 (PMC11547701; doi:10.3390/plants13212967)
Supplement: Supplementary file 1 [file plants-13-02967-s001.zip › Table S1.pdf]

Table S1. HPLC retention time (Rt), maximum absorption (λmax) and m/z of the identified major bioactive compounds from *Opuntia stricta* var. *Dillenii* based on Gómez-Lopez *et al.* [9].

Peak*	t_R (min)	Compounds	UV λmax (nm)	$[M-H]^+$	$[M-H]^-$	MS/MS (*m/z*)
1	7.880	Piscidic acid	272		255	193, 165, 135, 119, 107
2	9.947	Betanin	535	551		390, 389
3	14.176	Isobetanin	535	551		390, 389
4	24.171	Betanidin	538	389		345, 150
5	25.049	6´-O-sinapoyl-O-gompherin	539		755	225
6	27.186	2´-O-apiosyl-4-O-phyllocactin	537		767	551
7	25.140	5´´-O-E-sinapoyl-2´-apyosil-phyllocactin	248,330,540		975	---
8	30.410	Neobetanin	467		549	387
9	34.610	Quercetin-3-O-rhamnosyl-rutinoside (QG3)	358	757		611, 303
10	38.184	Quercetin glycoside(QG1) - Quercetin hexosyl pentosyl rhamnoside	255, 358	426		303, 191, 120
11	38.731	Quercetin glycoside(QG2) - Quercetin hexose pentoside	255, 353	653		303, 177
12	39.677	Isorhamnetin glucoxyl-rhamnosyl-rhamnoside(IG1)				
13	42.182	Isorhamnetin glucoxyl-rhamnosyl-pentoside(IG2)	254, 356	757		317, 167, 86

All characterization was done on a previous study (Gomez-Lopez et al; 2021a)
* Peak numbers are according to Figure S1
