# Supplementary material for: Effects of Opuntia stricta var. dillenii Extracts Obtained from Prickly Pear and an Industrial By-Product on Maturing Pre-Adipocytes
Source: Plants (Basel). 2024 Oct 24;13(21):2967. doi: 10.3390/plants13212967 (PMC11547701; doi:10.3390/plants13212967)
Supplement: Supplementary file 1 [file plants-13-02967-s001.zip › Table S2.pdf]

**Table S2**. Bioactive compounds content (mg/g dry weight) of the major betalains and, polyphenols, in peel, pulp and whole fruit and by-product (bagasse) from *Opuntia stricta* var. *dillenii* prickly pear based on Gómez-López *et al.* [9].

| Peak* | Compounds | Family | *Opuntia stricta var. dillenii*'s tissues | | | |
|---|---|---|---|---|---|---|
| | | | **Whole Fruit** | **Peel** | **Pulp** | **Bagasse** |
| | | | **mg/g dry weight** | | | |
| 1 | Piscidic acid | Phenolic acid | 1.64 ± 0.09[b] | 2.33 ± 0.33[a] | 0.62 ± 0.05[c] | 1.54 ± 0.05[b] |
| 2 | Betanin | Betalain | 2.97 ± 0.01[a] | 2.99 ± 0.05[a] | 2.91 ± 0.23[a] | 0.84 ± 0.02[b] |
| 3 | Isobetanin | Betalain | 1.85 ± 0.00[b] | 1.65 ± 0.04[b] | 2.28 ± 0.19[a] | 0.77 ± 0.02[c] |
| 4 | Betanidin | Betalain | 0.04 ± 0.00[a] | 0.04 ± 0.00[a] | 0.04 ± 0.01[a] | 0.02 ± 0.00[b] |
| 5 | 6´-O-sinapoyl-O-gompherin | Betalain | 0.13 ± 0.00[b] | 0.14 ± 0.00[a] | 0.08 ± 0.00[c] | 0.01 ± 0.00[d] |
| 6 | 2´-O-apiosyl-4-O-phyllocactin | Betalain | 1.29 ± 0.02[a] | 1.22 ± 0.02[a] | 1.61 ± 0.18[a] | 0.58 ± 0.16[b] |
| 7 | 5´´-O-*E*-sinapoyl-2´-apyosil-phyllocactin | Betalain | 3.14 ± 0.00[a] | 3.23 ± 0.13[a] | 2.60 ± 0.07[a] | n.d. |
| 8 | Neobetanin | Betalain | 1.95 ± 0.02[b] | 0.82 ± 0.00[d] | 3.26 ± 0.05[a] | 1.03 ± 0.07[c] |
| 9 | Quercetin-3-O-rhamnosyl-rutinoside (QG3) | Flavonoid | 0.04 ± 0.00[b] | 0.07 ± 0.00[a] | n.d. | 0.02 ± 0.00[c] |
| 10 | Quercetin glycoside(QG1) - Quercetin hexosyl pentosyl rhamnoside | Flavonoid | 0.04 ± 0.00[b] | 0.08 ± 0.00[a] | n.d. | 0.02 ± 0.00[c] |
| 11 | Quercetin glycoside(QG2) - Quercetin hexose pentoside | Flavonoid | 0.02 ± 0.00[a] | 0.02 ± 0.00[a] | n.d. | n.d. |
| 12 | Isorhamnetin glucoxyl-rhamnosyl-rhamnoside(IG1) | Flavonoid | 0.02 ± 0.00[a] | 0.03 ± 0.00[a] | n.d. | 0.01 ± 0.00[a] |
| 13 | Isorhamnetin glucoxyl-rhamnosyl-pentoside(IG2) | Flavonoid | 0.29 ± 0.00[b] | 0.52 ± 0.02[a] | 0.05 ± 0.00[d] | 0.18 ± 0.01[c] |
| | **Total major betalains** | | 11.37 ± 0.02[b] | 10.08 ± 0.03[c] | 12.78 ± 0.48[a] | 3.24 ± 0.27[d] |
| | **Total major polyphenols (Pisicidic acid and flavonoids)** | | 2.06 ± 0.09[b] | 3.04 ± 0.02[a] | 0.67 ± 0.05[d] | 1.78 ± 0.06[c] |
| | - **Total major flavonoids** | | 0.42 ± 0.00[b] | 0.72 ± 0.02[a] | 0.05 ± 0.00[d] | 0.24 ± 0.01[c] |

Results were expressed as mean ± standard deviation (n = 3). This came from obtaining at least two independent extracts (n = 2) and performing by HPLC determinations of each time (n = 2). Superscript letters indicate statistically significant differences (p ≤ 0.05) between different OPD tissues. *n.d. No detected;* * peak according to Table S1 and Figure S1
