# Supplementary figures and images for: Effects of Opuntia stricta var. dillenii Extracts Obtained from Prickly Pear and an Industrial By-Product on Maturing Pre-Adipocytes
Source: Plants (Basel). 2024 Oct 24;13(21):2967. doi: 10.3390/plants13212967 (PMC11547701; doi:10.3390/plants13212967)

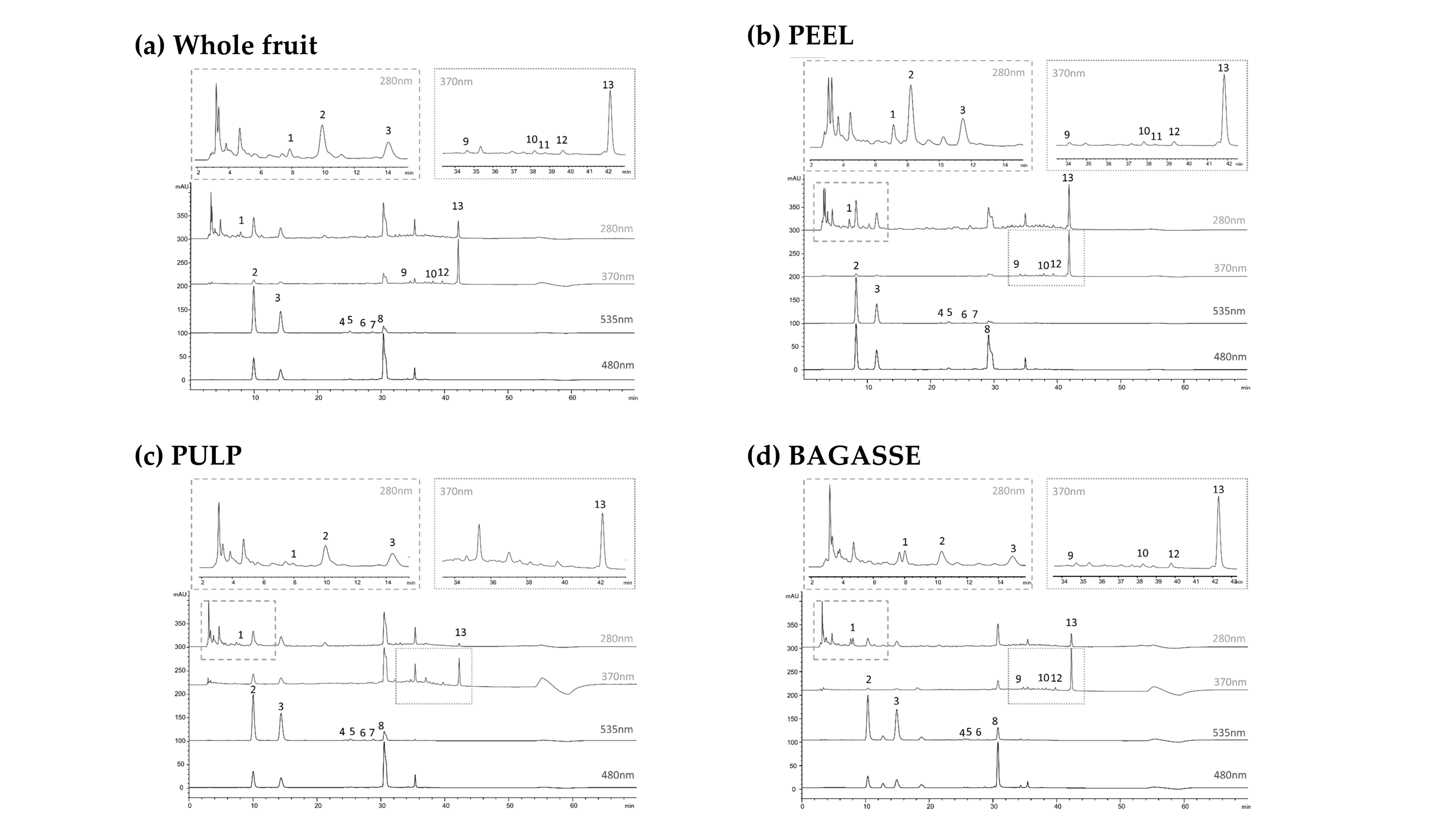

Supplement: Supplementary file 1 [file plants-13-02967-s001.zip › Figure S1.tif]
